# Adapting and implementing training, guidelines and treatment cards to improve primary care-based hypertension and diabetes management in a fragile context: results of a feasibility study in Sierra Leone

**DOI:** 10.1186/s12889-020-09263-7

**Published:** 2020-07-29

**Authors:** Guanyang Zou, Sophie Witter, Lizzie Caperon, John Walley, Kiran Cheedella, Reynold G. B. Senesi, Haja Ramatulai Wurie

**Affiliations:** 1 School of Economics and Management, Guangzhou University of Chinese Medicine, Guangzhou, China; 2 NIHR Research Unit on Health in Situations of Fragility, Institute for Global Health and Development, Queen Margaret University, Edinburgh, UK; 3 Nuffield Centre for International Health and Development, University of Leeds, Leeds, UK; 4 Royal College of General Practitioners, London, UK; 5 Directorate of Non-Communicable Diseases and Mental Health, Ministry of Health and Sanitation of Sierra Leone, Freetown, Sierra Leone; 6 NIHR Research Unit on Health in Situations of Fragility and College of Medicine and Allied Health Sciences, University of Sierra Leone, Freetown, Sierra Leone

**Keywords:** Non-communicable diseases, Primary care strengthening, Sierra Leone, Feasibility assessment, Fragile setting

## Abstract

**Background:**

Sierra Leone, a fragile country, is facing an increasingly significant burden of non-communicable diseases (NCDs). Facilitated by an international partnership, a project was developed to adapt and pilot desktop guidelines and other clinical support tools to strengthen primary care-based hypertension and diabetes diagnosis and management in Bombali district, Sierra Leone between 2018 and 2019. This study assesses the feasibility of the project through analysis of the processes of intervention adaptation and development, delivery of training and implementation of a care improvement package and preliminary outcomes of the intervention.

**Methods:**

A mixed-method approach was used for the assessment, including 51 semi-structured interviews, review of routine treatment cards (retrieved for newly registered hypertensive and diabetic patients from June 2018 to March 2019 followed up for three months) and mentoring data, and observation of training. Thematic analysis was used for qualitative data and descriptive trend analysis and t-test was used for quantitative data, wherever appropriate.

**Results:**

A Technical Working Group, established at district and national level, helped to adapt and develop the context-specific desktop guidelines for clinical management and lifestyle interventions and associated training curriculum and modules for community health officers (CHOs). Following a four-day training of CHOs, focusing on communication skills, diagnosis and management of hypertension and diabetes, and thanks to a CHO-based mentorship strategy, there was observed improvement of NCD knowledge and care processes regarding diagnosis, treatment, lifestyle education and follow up. The intervention significantly improved the average diastolic blood pressure of hypertensive patients (*n* = 50) three months into treatment (98 mmHg at baseline vs. 86 mmHg in Month 3, *P* = 0.001). However, health systems barriers typical of fragile settings, such as cost of transport and medication for patients and lack of supply of medications and treatment equipment in facilities, hindered the optimal delivery of care for hypertensive and diabetic patients.

**Conclusion:**

Our study suggests the potential feasibility of this approach to strengthening primary care delivery of NCDs in fragile contexts. However, the approach needs to be built into routine supervision and pre-service training to be sustained. Key barriers in the health system and at community level also need to be addressed.

## Background

Non-communicable diseases (NCDs), including heart disease, stroke, cancer, diabetes and chronic lung disease, contribute to almost 70% of all deaths worldwide [1]. More than 75% of all NCD deaths occur in low- and middle-income countries (LMICs). Each year, 15 million people die prematurely from an NCD, and 85% of these deaths occur in LMICs. The World Health Organisation (WHO) has proposed a framework for integrating NCD prevention into primary health care through a Package of Essential Noncommunicable (PEN) Disease Interventions for Primary Health Care in Low-resource Settings with a set of cost-effective priority interventions for poor-resource settings [2].

As the first point of contact with health services, primary-health-care (PHC) facilities are recognised as the most appropriate places for patient screening and early disease detection, continuous care provision for uncomplicated patients and referral of patients to specialists. Prevention and high-quality patient management are essential components in the control of NCDs such as hypertension and diabetes, and there is wealth of evidence on effective and cost-effective interventions for preventing and managing NCDs [3]. However, finding ways to implement these interventions and sustainably incorporate them into practice remains a challenge [3], especially in fragile settings. In sub-Saharan Africa, task-shifting, to overcome the shortage of trained physicians and other issues relating to access to primary care, is an increasingly widespread delivery approach for PHC interventions for hypertension, diabetes and other NCDs [4–7], along with other simple interventions [8, 9].

Sierra Leone, with a population of over seven million, has almost the lowest life expectancy at birth (52 years for men and 54 years for women) in the world [10]. The health system in Sierra Leone is also one of the most fragile in the world, partly due to a civil war between 1991 and 2002 that destroyed infrastructure and left thousands of people dead and displaced as refugees in neighboring countries (including the health workforce). Health systems in Sierra Leone were further burdened by the recent Ebola virus disease (EVD) outbreak in 2014 [11]. Sierra Leone is currently in a health system reconstruction phase.

While Sierra Leone is facing a high communicable disease burden, NCDs and its associated conditions represent an increasingly significant burden. WHO estimated that the percentage of deaths attributable to NCDs in Sierra Leone was 18% in 2008 and this has increased to 26% in 2012, with cardiovascular diseases accounting for 9% [12]. The WHO further estimated that around 30% of adult men and women had raised blood pressure respectively, while 4.8% of adults had raised blood glucose in 2014 [12]. Other risk factors of NCDs are also common: about 33% of men and 6.2% of women over 15 years smoked every day, and nearly 10 and 30% of adults were obese and overweight respectively [12]. Despite the increasing NCD burden, a scoping review in 2017 showed that the country’s capacity to address and respond to NCDs remained limited [13]. It highlighted that no specific programme or action plan was operational for the prevention and control of the major NCDs and risk factors. A more recent review of health system readiness for NCDs identified that NCD control receives very limited resources, with no NCD budget line, although both national and district stakeholders are increasingly aware of the importance of NCD control [14].

NCDs are still mainly being addressed at the tertiary care level, and patients often present at this level with complications of their uncontrolled disease. This highlights the need for context specific health education about NCDs and its associated complications and the need to strengthen the first point of contact within the health system – the PHC level. It is therefore important to explore how to improve the primary care-based risk reduction and case management for the prevention and control of the NCDs. Despite the introduction of the WHO PEN, this still needs to be operationalised in Sierra Leone [2]. However, a systematic review of community-based interventions for prevention of CVDs in LMICs suggested that training health care providers, implementing treatment guidelines and health education with a focus on diet and salt were key to the success of programmes of preventing CVDs [15].

With the NCD policy and strategic plan currently being reviewed, this study was timely as it could inform the review and implementation process in Sierra Leone. An earlier exploratory study highlighted a number of challenges of implementing NCD control in Sierra Leone, including financial barriers for users, lack of access to quality-assured drugs and high recourse to private and informal care seeking. However, it also identified potential leverage points for strengthening the system within existing (low) resourcing, such as through improved clinical guides and tools, combined with more effective engagement with communities, alongside regulatory and fiscal (tax revenue) measures [14]. Informed by this and other studies, an international partnership was developed to adapt and pilot guidelines and other clinical support tools to strengthen primary care-based hypertension and diabetes diagnosis and management in Bombali district, Sierra Leone. The aim was to develop learning to enable more effective primary care based NCD management in Sierra Leone and similar contexts. This study describes this intervention and assesses its feasibility.

## Methods

### Study setting

Bombali District is one of fourteen districts of Sierra Leone, located in the Northern Province, which borders the Republic of Guinea to the north. It is the second largest district (7985 km^2^) in Sierra Leone and had a population of 606,183 in 2015. The district has 19 Community Health Centres (CHCs), 36 Maternal and Child Health Posts (MCHPs), and 51 Community Health Posts (CHPs) at the primary care level; and two government hospitals, two private clinics. Local health services are managed by a District Health Management Team (DHMT). Each CHC is managed by a community health officer (CHO) and staffed with State Enrolled Community Health Nurses (SECHN) and Community Health Assistants (CHAs). The CHPs and MCHPs are managed by SECHNs and MCH Aides respectively.

### The partnership

The intervention was a partnership by a number of local and international organisations. The Royal College of General Practice (RCGP) came to Sierra Leone in 2017 to agree with Ministry of Health and Sanitation (MoHS) on developing an intervention based around training of CHOs on NCDS in Bombali District. This would be delivered and facilitated by 3 volunteer General Practitioners (GPs) from the UK working through Volunteer Service Overseas (VSO) as the local implementing partner. The package of tools was adapted from an earlier version developed by Communicable Diseases/Health Services Delivery Research Consortium^1^, University of Leeds, with support from the National Institute for Health Research (NIHR) funded Research Unit for Health In Fragility (RUHF) project,^2^ led by Queen Margaret University, Edinburgh, and working in partnership with the College of Medicine and Allied Health Sciences (COMAHS) at the University of Sierra Leone. Overall leadership came from the MoHS, while support to adaptation and pilot implementation came from the RCGP and VSO, working with the DHMT in Bombali. The RUHF project team led on the assessment of the intervention. The project has gone through three phases over March 2018 to June 2019: (1) intervention adaptation and development, (2) delivery of training and implementation of care improvement package and (3) collecting data to assess feasibility (Table 1).

**Table 1:** Components of intervention to improve primary care hypertensive and diabetic management in Bombali district, Sierra Leone

Training	- 35 CHOs were trained in group sizes of 10–20. The majority completed 4 out of 4 training modules, held on separate days and spread out over several months - 1 day for Midwives, SECHNs, MCH Aides
Equipment and materials	- Digital BP machine, glucometer, desktop guides, education picture card, eye test and BMI charts, and treatment cards
Mentorship	- Doctor and CHO mentoring visits to each CHC reviewing challenges, treatment cards, and clinical scenarios

### Data collection

The feasibility assessment includes understanding the processes of (1) intervention adaptation and development, (2) delivery of training, (3) implementation of care package and (4) preliminary outcomes of the intervention. The preliminary outcomes included improvement of systolic blood pressure (SBP) and diastolic blood pressure (DBP) after 3 months’ follow-up as compared to the baseline SBP and DBP level. The baseline BP level is the BP level recorded in the treatment cards at first consultation. A mixed-method approach, through mainly qualitative methods, was used. Table 2 lists the methods, key questions or indicators and time points of data collection in relation to the specific assessment questions.

**Table 2:** Methods of feasibility assessment

Research objectives	Methods	Key questions/indicators	Assessment time points
To document the processes of intervention adaption and development	Interviews with stakeholders	Working group processes, stakeholder engagement, outputs (e.g. desk guide)	During adaptation
	Participant observation		
To understand the processes of training delivery	Training register	Number of participants, training coverage	During and/or after the training
	Observation	Content, approach, effect of training	
	Interviews with stakeholders	Content, approach, effect of training	
To understand the processes of pilot implementation of the care improvement package	Treatment cards	Number and type of patients registered, doctors’ prescriptions, adherence to medication, lifestyle education	After pilot implementation
	Interviews with stakeholders	Factors related to provider and patients’ adherence to intervention	Before and after pilot implementation
	Mentoring/ supervisory visit reports	Progress and challenges	During pilot implementation
To assess preliminary outcomes of the intervention	Treatment cards	SBP, DBP	Endpoint of pilot implementation

#### Semi-structured interviews

Semi-structured interviews were conducted by researchers before and after the intervention. Interviewing sought to collect information related to the context of intervention, processes of intervention adaptation, and the respondent’s experiences, perspectives and opinions of the intervention (training and case management). In the first round of interviews, we purposively selected four CHCs, two in urban settings and two in more rural areas. Purposive sampling techniques were used to identify interviewees. In each CHC, we interviewed one CHO, two SECHNs. We selected 2 patients - one with hypertension and one with diabetes from each CHC. In addition, we interviewed one DHMT member who was involved with the intervention, and two doctors supporting it. In total, we conducted 23 and 28 interviews before and after the intervention respectively. We stopped the interviews when data reached a saturation point to address the research questions. The interview guide was specifically developed for this study, and has not previously been published elsewhere (Additional file 1).

The interview was conducted by experienced qualitative researchers. The interview with providers was conducted in English while an interpreter who was a recent public health graduate at University of Makeni, in Bombali district helped with the local language interviews. The interviews were recorded with consent from the participants. Each interview lasted between 30 and 45 min. Written consent was sought from all the interviewees.

#### Review of project reports

Training registers and training report forms were analysed to understand who attended or did not attend the training.

Records of mentoring and supervisory visits (by doctors together with the selected CHOs) were extracted and analysed to understand the project progress and challenges during the initial period and final period of implementation.

#### Observations

Participant observations were made during the intervention adaptation and development processes. Observations were conducted of all the training events by the doctors and researchers (if available for field visits), using a structured observation checklist. Observation was also conducted during the mentoring and supervisory visits by the doctors and researchers, using a structured observation checklist. The observational records were extracted and analysed during the initial period and final period of implementation.

#### Review of routine data (treatment cards)

The treatment registers and treatment cards were completed by CHOs on a routine basis based on the individual patients’ conditions. Review of treatment registers and cards helped to understand the diabetic and hypertensive control outcomes and case management information, such as prescription and use of related drugs and life- style interventions. In this paper we included the hypertensive patients who were recruited after the first training was conducted in May 2018 until 30 March 2019, and we defined the follow up period for all the included patients to be 3 months.

### Data analysis

Interview data was transcribed and framework analysis was used for qualitative data, starting from a coding frame based on the interview guides and study protocol, with new codes added inductively after reading through the transcripts. The themes initially included process of adaptation and development of intervention, delivery of the training and care improvement package, and factors influencing them (enablers and barriers to the intervention). Software (NVivo) was used to assist the data analysis.

Quantitative data was input into the Excel sheet and then exported to the SPSS 20.0. Descriptive analysis of quantitative data was carried out using mean, frequencies and percentages. Specifically, we conducted descriptive trend analysis for the follow up rates of hypertensive patients, and the SBP and SDP changes among the hypertensive patients on the monthly basis. T-tests were used to compare the SBP and DBP levels at baseline and three-month follow up among the hypertensive patients. Hypothesis testing was two-tailed, at the 5% level.

Data from different sources were integrated during analysis and write-up stages, either for supplementing or triangulating purposes under the main themes of the study.

## Results

This section reports the processes of intervention adaptation and training, findings on delivery of care delivery and health systems factors influencing the intervention and its feasibility.

### Intervention adaptation and development

Technical working groups (TWG) were established at local and national level in April 2018. The local TWG worked on the initial adaptation of the desktop guidelines for clinical management and lifestyle interventions, and associated training curriculum and modules, which had been pilot tested and shown to be acceptable and feasible in rural settings in China [16], Pakistan [17–19], Swaziland and other LMIC countries. The local TWG included two RCGP doctors and 5 CHOs who scored highly in the first module and were most keen to teach on NCDs and these CHOs became the trainers in the following training activities and mentors during the pilot implementation of the intervention.

The local TWG reviewed the materials and adapted each session to be context-specific and easy to use for CHOs during training and in the consultation room. Adaption also took into consideration recent evidence (e.g., WHO PEN), local terminology, and low availability of free, good quality, affordable and accessible diagnostics and medications. The local TWG kept regular interactions and communication with the national TWG, which included the NCD Directorate of MoHS, tertiary hospital NCD experts, so that the national needs and priority were fed into the adaptation processes. For instance, initially the adaptation was only targeted at CHOs, and at the request of the NCD Directory, the materials were also adapted for the lower level of health staff such as SECHNs, CHAs, MCH Aides. The national TWG experts reviewed the materials and a roundtable meeting was called to discuss and agree on the materials before the approval by MoHS.

The adaptation process lasted for 8 months between April and December in 2018. The national TWG approved a set of materials including (1) a guidelines (a desktop guide for use in clinics which gives staff accessible and practical advice about the diagnosis, management and follow-up care actions that need to be taken) that if feasible would be rolled out nationwide; (2) training materials for CHOs and other Primary Health Units (PHU) staff; (3) a treatment card (to improve management and follow up of NCDs); (4) eye test and BMI charts and a pictorial lifestyle change education chart.

Our interviews with the participants suggested that TWG allowed for a lot of learning within the group and developed members’ knowledge and confidence in NCDs significantly. The process guided major adaptions to make the materials more applicable to CHOs and their background knowledge and low resource setting and made it locally owned. The local TWG members met after the first time each module had been taught and they trained other groups as facilitators under this module. It was challenging to re-orient members from their accustomed lectures to a case study and role-play active learning approach. However, the training process helped identify problems and refine the desk-guide and training materials.

### Delivery of training

In total, 35 CHOs (community and hospital based) in the district had the opportunity to attend 4 modules on NCDs, each one on separate days (4 days), spread over several months. The three full day modules focused on communication skills, diagnosis and management of hypertension and diabetes, and the half day module included epilepsy and depression with a short session on cascading knowledge to other PHU staff (midwives, SECHNs and MCH Aides). For the full day modules, the CHOs were split into two groups (15 for each group approximately). The first training in each full day module served as the pilot course that helped to refine the desk-guide and training materials prior to subsequent training of the remaining groups. The hospital CHOs, who are responsible for primary care to a local population, were also trained to ensure consistent and appropriate NCD management at all levels and stages of healthcare. The final half day module was done as one large group.

In addition, approximately 300 PHU staff (midwives, SECHNs and MCH Aides) attended a one-day training course, facilitated by the trained CHOs with support from the RCGP/VSO doctors. This included sections on communication skills, lifestyle planning, and diabetes and hypertension basics. This aimed to increase detection, referral, and lifestyle behaviour change (but not to initiate prescription of a medication or open a treatment card, which was done by CHOs).

The training improved the knowledge of hypertension and diabetes among CHOs and other PHU staff, as suggested by the pre-post tests conducted by the RCGP/VSO doctors (Table 3). The training provided was seen as helpful by all health workers interviewed. They valued the knowledge they were taught and reported that they had put it into use since.

*‘It [the training] went well because at times when we are choked up with job, when we see the patient after medication, we now give them lifestyle advice. … We cannot do everything all by ourselves, we share the responsibilities with other health workers who also had the training’ (CHO Manager 2).*

**Table 3:** Pre-post test result of knowledge of hypertension and diabetes among training participants

	CHO Module 1 Group A^a^	CHO Module 2		CHO Module 3		Other PHU staff	
		Group A	Group B	Group A	Group B	Group A	Group B
Pre-	42%	51%	56%	63%	60%	53%	50%
Post-	53%	60%	65%	70%	69%	68%	68%

^a^Group B test results were missing

Almost all health workers complained that the training was too short and called for more training on NCD prevention. It was acknowledged that training all health workers was a vast undertaking, given the amount of time taken to explain all points and the low starting levels of knowledge on the recognition and management of hypertension and diabetes and the very limited teaching during CHO training (which covers a broad range of medicine and surgery over a short 3-year training period.) Providing messages/training via TV was suggested as an alternative for refresher training, but it was acknowledged that this might not be easy due to unreliable electricity supplies and lack of television equipment in health facilities. The findings also raise the importance of improved pre-service training in NCDs for frontline clinical staff like CHOs.

### Processes and preliminary outcomes of implementing the care improvement package

#### NCD diagnosis and symptoms

Consistent with the improved scores after training, the CHOs reported that training improved their confidence and skills in diagnosing NCD patients and they demonstrated some knowledge of hypertension and diabetes diagnosis. For instance, CHOs and other PHU staff (SECHNs and MCH Aides) were aware of the importance of doing two blood pressure tests for diagnosis. However, their capacity for case identification was poor, given the low number of cases identified (based on our observation and interviews).

Our interviews suggested that the numbers of patients identified with hypertension and diabetes averaged about 2 per CHC (ranges from 0 to 12), which is probably mainly due to low attendance rates at clinics. Health workers stated that NCD patients were being more widely diagnosed than before the intervention, marking an improvement in practice and awareness. According to our interviews, the number of hypertension patients diagnosed in CHCs varied – the diagnosed numbers were: 9 (with one transferred to Freetown), 11, 12, 10 and one CHC reported over 60 patients since the intervention started (until feasibility assessment interviews were conducted in June, 2019). The number of diabetes patients diagnosed in CHCs varied but was lower than the number of hypertensive patients diagnosed. The numbers CHOs stated were: 0, 2, 0, 0, 1. CHOs reported lack of working equipment to diagnose high blood glucose levels.

Analysis of the treatment cards suggested similar results. From June 2018 to March 2019, 50 (94%) of 53 treatment cards retrieved were recorded as having hypertension (including 19 male and 31 female patients, average age 62), while only 3 (6%, male, average age 59) were recorded as having diabetes.

#### Desktop guides and treatment cards

Desk guides were observed in use in two of the four CHCs visited, and CHOs stated that they were useful. As a CHO commented:

*‘Before now, we looked in books, it took more time, but now we only need to look at the technical guidelines and flow charts.’* CHO

Treatment cards and patient registers were reported as being widely used in the four CHCs visited. One CHO suggested that the treatment cards could be revised to be more concise. Another, on the other hand, preferred to use a notebook to list patient details rather than the treatment cards because he could record more information there. Though treatment cards were being used, our interviews and observation suggested that most CHCs recorded fewer treatment cards than NCD patients. Some CHOs need more encouragement to complete forms. One CHO stated that they only used treatment cards when they prescribed drugs to the patient, which was not their intended use (they should be used for all cases).

#### Compliance with medication

**Fig. 1:**
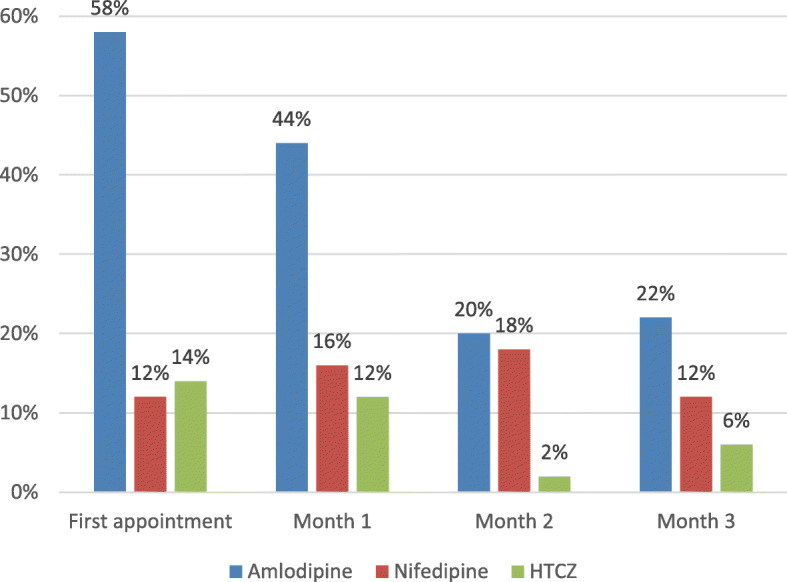
Top three medications prescribed for hypertensive patients

Analysis of the treatment cards suggested that the three most often prescribed hypertensive drugs were Amlodipine, Nifedipine, and hydrochlorothiazide (HTCZ) during 3 months’ follow up period (Fig. 1). Although Amlodipine remained the most often prescribed, the prescription rate declined consecutively from the first consultation (58%) to Month 1 (44%), Month 2 (20%) and Month 3 (22%).

Some patients who were interviewed (5 of 7 interviewed) stated that they had been taking their medication to manage their condition:

*‘When I give money they give me the medicine, only the hypertension medicine they’re giving me. Like yesterday I spent le50.000 and they give me the hypertension medicine that I should take throughout the month [until the next appointment].” Patient with hypertension.*

Due to financial constraints, health workers reported that many patients could not afford to comply with instructions to take medication regularly to treat their NCD conditions (e.g. hypertension).

#### Lifestyle advice

All health workers interviewed showed some awareness of the importance of lifestyle advice for those suffering from hypertension and knowledge of how to treat patients with NCDs, including how to deliver lifestyle advice, indicating this aspect of the intervention had been effective. Many CHOs gave advice to patients to reduce their unhealthy lifestyle habits bit by bit, encouraging them not to give up suddenly, but to gradually improve their lifestyle. As showed by the treatment card analysis, the most common lifestyle messages for patients were exercise and low salt. CHOs reported that some patients say they have made lifestyle changes, but contrasting signs were sometimes noted (for example, patients claiming they have given up smoking but still smelling of smoke).

#### Follow up management

**Fig. 2:**
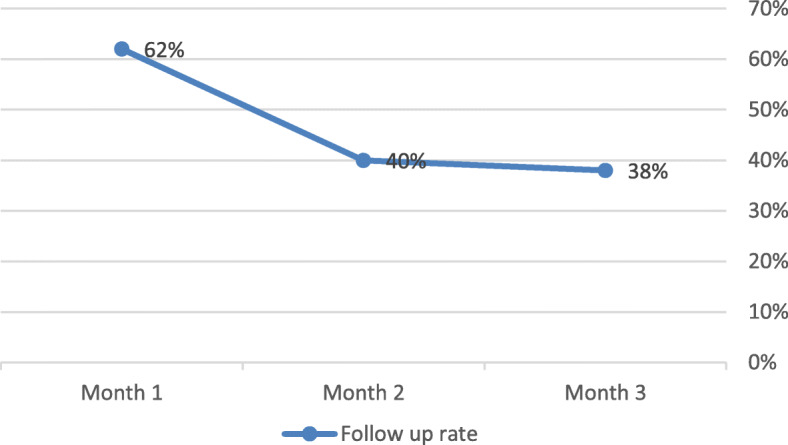
Follow up rates among hypertensive patients

Patients reported that once they had been diagnosed, they did attend the CHC for follow-up. However, connected to distance to health facilities and lack of mobility, health workers and patients reported that lack of follow up was a common problem. It was common that patients did not return for further treatment for NCDs due to the cost of transport or medication. Some patients did not go to their follow up appointment because they started to feel better when they start taking medication. Several patients went to hospital to treat acute symptoms when regular follow up at their health centres could have prevented this. Many CHOs stated that the only sure way to follow up with patients was to visit their homes. However, this was not practical for many health workers, though community health workers (CHWs) were discussed as a potential resource who could regularly visit patients’ home due to their close proximity (often living in communities). The treatment card analysis showed that of 50 hypertensive patients recruited for evaluation, the proportion of the hypertensive patients who attended the follow up appointment reduced from 62% in Month 1 to 40% in Month 2 and 38% in Month 3 (Fig. 2).

#### Referral

CHOs stated that they thought referral processes were not always working. Often patients could not afford to travel to the hospitals they were referred to. CHOs reported that they had not referred many patients to hospitals.

#### Mentoring and feedback

Most CHCs received a mentoring visit conducted by local TWG members. The intention was to develop a peer support process which would continue longer term. During the mentoring visits to the CHCs, mentors acted as colleagues supporting other CHOs to improve the quality of care, by assessing treatment activity, identifying problems and solutions, building confidence and skills and encouraging more screening. All 22 CHCs were visited by the end of April, 2019. Mentors completed and submitted the mentoring report based on a standard mentoring form. CHOs agreed that the mentoring they received was very helpful. There was also a WhatsApp group to share information and advice about individual clinical cases between CHOs and VSO doctors.

From the mentoring the TWG had done, they noticed there was misdiagnosis and mis-treatment of NCDs, and feedback was given during the visit. There are widespread mistaken beliefs about hypertension held by both health staff and the community. It was seen as an acute, dangerous symptomatic disease, which affects what type of treatment is expected for it. Overtreatment has continued in the hospital despite training provision. The mentoring visits also found that some patients in PHUs were given the available but unsuitable medicines such as methyldopa, meant for pregnant women through the free health care initiative.

#### Preliminary outcomes

**Fig. 3:**
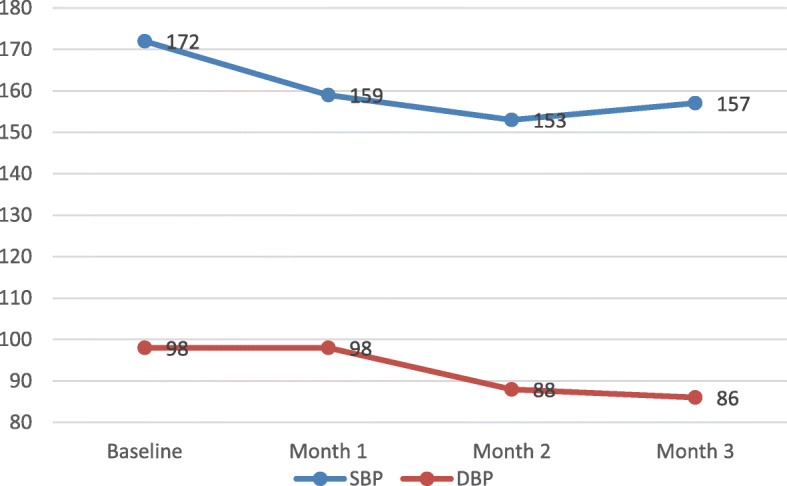
SBP and DBP among hypertensive patients

The treatment card analysis showed a positive outcome after initiation of treatment for hypertension (Fig. 3). The SBP for all the hypertensive patients (*n* = 50) steadily decreased from baseline (172 mmHg) to Month 1 (159 mmHg) and Month 2 (153 mmHg), before increasing again in Month 3 (157 mmHg). The average SBP decreased by 15 mmHg from baseline to Month 3, which is not statistically significant (t = 1.701, *p* = 0.106). The DBP for all the hypertensive patients remained unchanged from baseline (98 mmHg) to Month 1 (98 mmHg) then decreased dramatically in Month 2 (88 mmHg), and slightly in Month 3 (86 mmHg). The average DBP achieved a significant reduction by 12 mmHg from baseline to Month 3 (t = 4.069, *p* = 0.001).

### Health systems factors influencing the pilot implementation

Our interviews and observations identified a number of health systems factors that affected the pilot implementation of the care improvement package.

#### Distance to health facility

Some patients paid for transport to attend the health facilities, others used motorbikes, but several patients and CHOs stated that it was difficult for patients to get to the health facility and this affected follow up and referral.

#### Equipment not working or lacking

There were numerous accounts of equipment in the health facilities not functioning or not being maintained. A common example was blood pressure monitors not working or running out of battery, while no one at the CHC had money or time to replace them. Most CHCs did not have working glucometers and all CHOs stated they struggled to get access to glucose testing strips. Most CHCs had BP machines, but lacked a battery. Almost all health workers who were interviewed, called for more and improved equipment (e.g. BP machines, glucometers, strips for measuring blood glucose levels) as well as training on how to use it.

#### Lack of drug supplies

There was an overwhelming consensus that health facilities did not have enough drugs and those that they did have were not affordable. Interviews show that the drug supply (apart from the free MNCH drugs) is provided through ad hoc/informal practices, including purchasing and selling drugs to patients.

*‘Some of these drugs are not in the facility unless we buy them because they are not supplying them to us, so at least if they start giving them to us … we are really in constraint for the drugs in the facility unless we have to go to Makeni for them.’ MCH Aide.*

#### Financial barriers

Medication access and affordability has, as expected, been a major difficulty. Only a very small minority are likely to be willing or able to pay for two or more drugs per month. Many patients struggled to pay for the medication they needed after they were diagnosed with hypertension. This was an impediment to them getting adequate treatment for their condition. Many CHOs, SECHNs and MCH Aids stated there was a desperate need to provide affordable drugs to patients.

#### CHO-patient relationship

Both CHOs and patients reported trust in the CHO-patient relationship. Patients reported that speaking to the CHO made them feel better and that they felt comfortable speaking to the CHOs and other health workers.

#### Traditional healers

CHOs stated that traditional healers were important influencers in communities. Two CHOs stated that they had worked to develop friendly relationships with traditional healers. These CHOs had worked with traditional healers to help them understand the importance of them sending sick people to the CHC for medical treatment. This was being done with varying degrees of success.

Nevertheless, there were several examples given by health workers of traditional healers not managing NCDs conditions well in people they saw, allowing them to get worse, and eventually resulting in patients going to CHCs needing urgent medical attention with acute medical conditions, for example very high blood pressure. Often when they arrive at the CHCs their conditions (e.g. hypertension) are serious. This adds to the challenge of case management for the CHOs.

## Discussion

This is one of the first studies to describe the adaptation and implementation of interventions to improve primary care based hypertensive and diabetic management in a fragile setting and to assess its feasibility. Our study demonstrates that a partnership between the local and national health authorities, international NGOs and researchers could work to facilitate NCD service delivery improvement at primary care level in Sierra Leone. The pilot intervention improved the SBP and DBP in 3 months. It worked as intended in terms of process of implementing the care improvement package to some extent, thought numerous challenges in health systems hindered the effective delivery of intervention. A number of key issues arise from this study that will help to generate lessons for much-needed future NCD interventions in fragile settings.

### Technical working group process

In this partnership, the TWG has played a crucial role in planning, adapting, training, implementing and mentoring of the intervention throughout the process. With the TWG process, the project effectively engaged the local and national stakeholders to improve the content and chance of local ‘buy-in’ to the technical guidelines. As previous studies have suggested, TWG activities build local research capacity, foster in-country ownership and promote research uptake [3, 20]. In LMICs, especially in fragile settings, service delivery in primary care settings for patients with NCDs is generally “unstructured” and poorly monitored. One study implementing a nurse-led NCD service in a resource-poor area of South Africa suggested the use of protocols and treatment strategies that were simple and responsive to the local situation enabled the majority of patients to receive convenient and appropriate management of their NCD at their local primary care facility [21]. The TWG developed a package, which includes simple, systematic, user-friendly, and context-specific case management guides and supporting tools for training and service performance monitoring at primary care. This package has potential to promote quality and consistency of NCD care at primary care. The materials could be further adapted and incorporated into the national plan and national curriculum for the pre-service training programme for CHOs and other cadres, such as nurses. The TWG members acted as trainers and mentors, which was found to be an effective peer support strategy for the intervention implementation phase.

### Process of implementing the care improvement package

A systematic review and evidence synthesis of primary care approaches for chronic disease in Sub-Saharan Africa suggested that the use of standardized protocols for diagnosis, treatment, monitoring and referral to specialist cares should become one of the priorities for disease management at primary healthcare clinics [22]. Our study highlighted mixed results of implementing the NCD care improvement package in terms of diagnosing, treating, conducting lifestyle education and follow up, using desktop-guides and treatment cards and other supportive materials, at the primary care level in a fragile context. On the one hand, these improved processes may have helped to achieve positive improvement of SBP and DBP in 3 months. On the other hand, despite the reported improvement in NCD knowledge and care processes, case identification, use of standard guidelines and tools (e.g., treatment cards), treatment capacity and referral processes remained sub-optimal. For instance, among the 53 eligible treatment cards for hypertensive and diabetic patients, only 3 were recruited as diabetic patients during the defined period. This indicates the very poor capacity of case detection for diabetes in primary care settings (as the prevalence of raised blood glucose was nearly 5% among the adults in 2014 [12]). Our results suggest that implementing the intervention in primary care requires strengthening of training and mentoring, both during pre-service training, and in-service training, to improve the diagnostic and treatment capacity and counselling skills.

### Addressing health systems and community barriers to improve the primary care delivery of NCDs

A systematic review of primary care approaches for chronic disease in sub-Saharan Africa suggested the importance of the availability of essential diagnostic tools and medications, in addition to the use of standardized care protocols for disease management at local primary healthcare clinics [22]. Consistent with our exploratory study [14], this study identifies a number of health systems barriers that are typical of fragile settings [23] and even wider sub-Saharan Africa [24–26] and which hinder the effective delivery of NCD care in primary care, such as distance, cost of transport and medications, lack of supply of medications, poor treatment equipment and health seeking behaviours which often delay care-seeking or prioritise traditional medicine. These barriers may have jointly contributed to the low primary care attendance of NCD patients and hence poor detection of hypertensive, and in particular diabetic cases,- which require more sophisticated diagnosis and treatment. CHCs need to be routinely seeing a large proportion of the adult population in the course of a year to detect most patients with asymptomatic conditions. In particular, lack of equipment or working equipment not only hinders the diagnosis but also the timely treatment and consistent follow-up care. Shortage of drug supply and financial barriers, as indicated by the decrease of Amlodipine, the most common prescription, prevent timely access to NCD care and quality of case management.

The barriers we identify occupy the often turbulent space between communities and the health system which have been shown to be problematic in fragile health settings [27]. As such, it is clear from our findings that further work needs to be done to strengthen weak public health systems in fragile health settings to enable them to more effectively interact with the needs of vulnerable communities, promoting engagement with all providers and communities, and building awareness and trust. CHOs will need on-going support, mentoring and essential NCD supplies, all of which requires small but critical funding. This can be done, for example, by ensuring interventions such as ours are properly embedded into a national NCD strategy which is supported to be scalable and sustainable. Similarly, greater communication should be stimulated between the health system and communities through, for example, social mobilisation or other community engagement activities around NCDs which are connected to the wider public health system. Such activities have been recently advocated [28, 29] and found to be valuable in tackling NCDs in other fragile health settings [30, 31].

## Limitations

This article reports on a pilot intervention project conducted in a district of Sierra Leone. The focus was on assessing feasibility, using mixed methods, with an emphasis on qualitative data, which has deeper exploratory potential. The qualitative data may be biased as respondents may have chosen to provide positive responses in the interviews, although we disseminated and validated the key messages in a district-wide CHO meeting. Given the data system limitations in Sierra Leone, quantitative sources also have weaknesses, which are balanced through our use of mixed methods. The mix of data sources, including observation and record checking, supports more robust overall assessment. In this study, sample size included for evaluation was too small (especially with only three diabetic patients recruited) and the follow up period may be too short to achieve the optimal BP change. In addition, we did not assess the incremental costs of the intervention, related to training, implementation and treatment (direct medical and non-medical costs such as transportation costs), given the focus of the research on feasibility. Long-term follow up of the intervention with a more rigorous evaluation design, including incremental cost-effectiveness analysis, would be an appropriate next step to better understand the effects and sustainability of the intervention.

## Conclusion

Our study demonstrates how an international partnership can work to adapt and support a primary care strengthening intervention for NCDs in a fragile setting. Our study suggests the potential feasibility of this intervention. However, careful attention needs to be paid to sustainability and integration of the approach in pre-service training, as well as addressing significant remaining barriers in the health system and at community level.

## Supplementary information

**Additional file 1.** Interview guides for RUHF feasibility study of strengthening NCD services in Sierra Leone.

## Data Availability

The datasets used and/or analysed during the current study are available from the corresponding author on reasonable request.

## Abbreviations

CHAs: Community health assistants
CHCs: Community health centres
CHPs: Community health posts
CHO: Community health officer
DBP: Diastolic blood pressure
DHMT: District health management team
HTCZ: hydrochlorothiazide
MCHPs: Maternal and child health posts
NCDs: Non-communicable diseases
PHU: Primary health unit
SBP: Systolic blood pressure
SECHNs: State enrolled community health nurses
WHO: World health organisation

## Footnotes

1 (http://comdis-hsd.leeds.ac.uk)

2 (https://www.qmu.ac.uk/research-and-knowledge-exchange/research-centres-institutes-and-knowledge-exchange-centres/institute-for-global-health-and-development/nihr-research-unit-on-health-in-fragility-ruhf/)

## References

[1] Noncommunicable diseases. https://www.who.int/news-room/fact-sheets/detail/noncommunicable-diseases. Accessed 30 Nov 2018.

[2] World Health Organisation (2010). Package of Essential Noncommunicable (PEN) Disease Interventions for Primary Health Care in Low-Resource Settings.

[3] Walley JD, Graham K, Wei X, Kain K, Weston R (2012). Getting research into practice: primary care management of noncommunicable diseases in low- and middle-income countries. Bull World Health Organ.

[4] Bello SI, Ganiyu KA, Dakop YO, Erah PO. Pharmacist's intervention in the control of blood sugar levels in randomised diabetes patients at a primary health care setting in Benin City. Nig Q J Hosp Med. 2012;22(4):245–8. 24568058

[5] Blackstone S, Iwelunmor J, Plange-Rhule J, Gyamfi J, Quakyi NK, Ntim M, Ogedegbe G: Sustaining Nurse-Led Task-Shifting Strategies for Hypertension Control: A Concept Mapping Study to Inform Evidence-Based Practice. Worldviews Evid Based Nurs 2017, 14(5**,** 1741–6787 (Electronic)). 10.1111/wvn.12230 28449387

[6] Kengne AP, Pk A, Fezeu LL, Sobngwi E, Mbanya JC. Primary health care for hypertension by nurses in rural and urban sub-Saharan Africa. J Clin Hypertens (Greenwich). 2009;11(10):564–72. 10.1111/j.1751-7176.2009.00165.x PMC8673012 19817937

[7] Labhardt ND, Balo Jr, Ndam M, Grimm JJ, Manga E: Task shifting to non-physician clinicians for integrated management of hypertension and diabetes in rural Cameroon: a programme assessment at two years. BMC Health Serv Res 2010, 10(1472–6963 (Electronic)). 10.1186/1472-6963-10-339 PMC3018451 21144064

[8] Kande C, Mash R (2014). Improving the quality of care for patients with hypertension in Moshupa District, Botswana: quality improvement cycle. Afr J Prim Health Care Fam Med.

[9] Labhardt ND, Jr B, Ndam M, Manga E, Stoll B. Improved retention rates with low-cost interventions in hypertension and diabetes management in a rural African environment of nurse-led care: a cluster-randomised trial. Tropical Med Int Health. 2011;16(10):1276–84. 10.1111/j.1365-3156.2011.02827.x 21733046

[10] WHO country office webpage. http://www.who.int/countries/sle/en/. Accessed 30 Nov 2018.

[11] McPake B, Witter S, Ssali S, Wurie H, Namakula J, Ssengooba F (2015). Ebola in the context of conflict affected states and health systems: case studies of northern Uganda and Sierra Leone. Confl Heal.

[12] Organization WH: Atlas of the African health statistics**.** 2017.

[13] Idriss A, Wurie HR, Bertone MP, Elimian K, Vidal N, Samai M: ncd-scoping-study. http://chwcentral.org/policy-community-health-workers-sierra-leone. 2018 Accessed 3 Dec 2018 2018.

[14] Witter S, Zou G, Diaconu K, Senesi RGB, Idriss A, Walley J, Wurie HR (2020). Opportunities and challenges for delivering non-communicable disease management and services in fragile and post-conflict settings: perceptions of policy-makers and health providers in Sierra Leone. Confl Heal.

[15] van DV, Oti S, Addo J, de Graft-Aikins A, Agyemang C (2012). Review of community-based interventions for prevention of cardiovascular diseases in low- and middle-income countries. Ethn Health.

[16] Wei X, Walley JD, Zhang Z, Zou G, Gong W, Deng S, Harries AD, Hicks JP, Chong MKC, Newell JN (2017). Implementation of a comprehensive intervention for patients at high risk of cardiovascular disease in rural China: a pragmatic cluster randomized controlled trial. PLoS One.

[17] Khan MA, Khan N, Walley JD, Khan SE, Hicks J, Sheikh FI, Khan MA, Ali M, Ahmed M, Khan HJ (2019). Enhanced hypertension care through private clinics in Pakistan: a cluster randomised trial. BJGP Open.

[18] Khan MA, Walley JD, Ali S, King R, Khan SE, Khan N, Sheikh FI, Khan HJ: Process evaluation of integrated diabetes management at primary healthcare facilities in Pakistan: a mixed-methods study. BJGP Open 2018, 2(4):bjgpopen18X101612. 10.3399/bjgpopen18X101612 PMC6348331 30723798

[19] Khan MA, Walley JD, Khan N, Hicks J, Ahmed M, Khan SE, Khan MA, Khan HJ, Harries AD (2018). Effectiveness of an integrated diabetes care package at primary healthcare facilities: a cluster randomised trial in Pakistan. BJGP Open.

[20] Wei X, Walley JD, Liang X, Liu F, Zhang X, Li R (2008). Adapting a generic tuberculosis control operational guideline and scaling it up in China: a qualitative case study. BMC Public Health.

[21] Coleman R, Gill G, Wilkinson D (1998). Noncommunicable disease management in resource-poor settings: a primary care model from rural South Africa. Bull World Health Organ.

[22] Kane J, Landes M, Carroll C, Nolen A, Sodhi S (2017). A systematic review of primary care models for non-communicable disease interventions in sub-Saharan Africa. BMC Fam Pract.

[23] Idriss A, Diaconu K, Zou G, Senesi RG, Wurie H, Witter S: Rural–urban health-seeking behaviours for non-communicable diseases in Sierra Leone 2020, 5(2):e002024. 10.1136/bmjgh-2019-002024 PMC7053783 32181002

[24] Nyaaba GN, Masana L, de-Graft Aikins A, Beune E, Agyemang C: Factors hindering hypertension control: perspectives of front-line health professionals in rural Ghana. Public Health 2020, 181(1476–5616 (Electronic)). 10.1016/j.puhe.2019.11.007 31923796

[25] Njuguna B, Vorkoper S, Patel P, Reid MJA, Vedanthan R, Pfaff C, Park PH, Fischer L, Laktabai J, Pastakia SD (2018). Models of integration of HIV and noncommunicable disease care in sub-Saharan Africa: lessons learned and evidence gaps. Aids.

[26] Chikowe I, Mwapasa V, Kengne AP: Analysis of rural health centres preparedness for the management of diabetic patients in Malawi. BMC Res Notes 2018, 11(1**,** 1756–0500 (Electronic)). 10.1186/s13104-018-3369-7 PMC5932777 29720279

[27] Diaconu K, Falconer J, Vidal N, O’May F, Azasi E, Elimian K, Bou-Orm I, Sarb C, Witter S, Ager A (2019). Understanding fragility: implications for global health research and practice. Health Policy Plan.

[28] Amminadab J: First-ever WHO global report on epilepsy highlights care gap in poorer countries | UN News, UN News,. Available at: HTTP://news.un.org/en/story/2019/06/1040901 (Accessed: 28 June 2019). ). In.; 2019.

[29] Heller O, Somerville C, Suggs LS, Lachat S, Piper J, Aya Pastrana N, Correia JC, Miranda JJ, Beran D. The process of prioritization of non-communicable diseases in the global health policy arena. Health Policy Plan. 2019. 10.1093/heapol/czz043 PMC6736081 31199439

[30] Dyson PA, Anthony D, Fenton B, Stevens DE, Champagne B, Li L-M, Lv J, Ramírez Hernández J, Thankappan KR, Matthews DR (2015). Successful up-scaled population interventions to reduce risk factors for non-communicable disease in adults: results from the international community interventions for health (CIH) project in China, India and Mexico. Plos One.

[31] Granada-Echeverri P, Zapata-Valencia CD, Giraldo-Trujillo JC: The impact of a social mobilisation model on promoting physical activity amongst people affiliated to the Colombian Social Health Security System.’, Impacto de un Modelo de Movilizacion Social sobre la promocion de la actividad fisica en afiliados al Sistema de Seguridad Social en Salud. Bogota, 10(3), pp. 361–373. doi: http://dx.doi.org**/**10.1590/S0124-00642008000300001. 2018. 10.1590/s0124-00642008000300001 19043627

