# Supplementary material for: Adapting and implementing training, guidelines and treatment cards to improve primary care-based hypertension and diabetes management in a fragile context: results of a feasibility study in Sierra Leone
Source: BMC Public Health. 2020 Jul 29;20:1185. doi: 10.1186/s12889-020-09263-7 (PMC7392674; doi:10.1186/s12889-020-09263-7)
Supplement: Supplementary file 1 — Additional file 1. Interview guides for RUHF feasibility study of strengthening NCD services in Sierra Leone. [file 12889_2020_9263_MOESM1_ESM.docx]

**Additional file 1 Interview guides for RUHF feasibility study of strengthening NCD services in Sierra Leone**

**Interviews before intervention is implemented**

**Patients**

1. Can you tell us about your family (how many members are there in your family, income, education, etc.)?
2. Which health conditions do you suffer from (hypertension and/or diabetes)? When did you find out you had hypertension/diabetes? How do you find out? Do you feel that you properly understand your disease (hypertension and/or diabetes)?
3. Are you seeking any care for hypertension and diabetes? Where do you seek care from (CHC, district hospital, traditional healers)? Why do you use these services? How effective do you think the treatment is that you receive?
4. Are you currently on any medication for hypertension and diabetes? Why or why not? If you are on medication, do you always take it, why or why not?
5. Can you tell me about your lifestyle (smoking, acholic use, salt intake, exercise)? Have you received any advice from your CHOs, traditional healers, Murryman or other people who provide you with counselling?
6. How much money do you spend on controlling your hypertension and diabetes? What do you spend money on to help you control it (e.g. transport)? Do you feel that it is money well spent? Why or why not? Can you afford what you spend on controlling it?
7. What do you expect from health services that provide you with care for your hypertension and diabetes?

**CHOs**

1. What is your role? What are your responsibilities? (including education and career history)
2. Did you receive any training on NCDs (e.g. hypertension and diabetes) before you worked as a CHO?
3. Were you involved with the VSO intervention adaptation, development, and care set up processes (treatment cards, drug supplies etc)? If yes, how? How do you feel about the intervention (e.g. the working group process)?
4. Did you receive training from the VSO doctors? What do you think about the model of training (pilot/training of trainers followed by training of remaining CHOs for each section), organisation, content and effect of the training?
5. Can you describe how you now diagnose patients (since this training)? How did you diagnose patients before the training?
6. Can you describe how you manage and follow up patients? (prescription, lifestyle, adherence support, referral, etc) [This question was explored in April, and will be further explored, if time permitted]
7. What are your expectations and suggestions to improve the guidelines, training materials and intervention?

**SECHN**

1. What is your role? What are your responsibilities? (including education and career history)
2. Can you describe your everyday work in the CHCs? Do you counsel NCD patients? How?
3. Have you received any training on NCDs? When and where, and how?
4. What do you think you can help to improve NCD control as a SECHN? Is it feasible for you to better educate NCD patients? Is it feasible for you to follow up these patients (e.g. by phone, home visits)?

**DHMT member**

1. Were you involved with the intervention adaptation, development and care set up processes (treatment cards, drug supplies etc)? If yes, how were you involved? How do you feel about the processes in the implementation of the intervention (e.g. the working group process)?
2. What is your impression of the project so far (progress, fidelity, quality)? What are the reasons for these impressions? (barriers and enablers)
3. What do you think about the model (pilot/training of trainers followed by training of remaining CHOs for each section), organisation, content and effect of the training?
4. What needs to be improved in terms of guidelines and training materials？
5. What are your suggestions for sustaining and scaling up this approach?

**VSO/RCGP doctors**

1. How do you feel about the process of intervention adaptation, development and the care set up processes (e.g. treatment cards, drug supplies)? What are the enablers and barriers?
2. What do you think about the model of training (pilot/training of trainers followed by training of remaining CHOs for each section), organisation, content and effect of the training?
3. What needs to be improved in terms of guidelines and training materials, organization and delivery of intervention?

**Interviews after intervention has been implemented**

**Patients**

1. Please can you tell me about the state of your health (hypertension and/or diabetes)?
   1. Do you feel that you properly understand your disease (hypertension and/or diabetes)
   2. Do you feel that you understand why you have had tests (e.g. blood pressure, blood tests)?
2. About communications with the CHO
   1. Do you understand why you are having these consultations?
   2. Do you feel that you have enough time to ask questions about your disease and treatment?
   3. What are the main problems you feel when you talk with CHOs?
3. Are you prescribed with any drugs? Where do you get them, CHC or pharmacy?
4. Are you taking these medicines? Why or why not? Do you understand or have you experienced any potential side effects from the prescription?
5. Do you undertake the following:
   1. follow-up appointments;
   2. taking regular medication;
   3. strategies used to support adherence (SMS messages, treatment supporters)? Why do you think these are being used / not used?

Can you suggest any other ways to support you in adherence?

1. Have you received any information about changing your lifestyle (drug compliance, attendance, diet, smoking cessation)? Give details about what you have been told, including information and strategies for making changes to your lifestyle.
2. Have you made any changes in your lifestyle following intervention? Why / why not? Which changes have you made and why? Do you think that you can adhere to these changes? Why/why not?
3. Costs of attending consultations:
   1. How much do you spend? What do you spend it on (e.g. transport)?
   2. Do you feel that it is money well spent? Why or why not?
   3. Can you afford it?
   4. Are there any other factors that influence your visit to the CHC? If yes, what are they?
4. Is there anything else you would like to tell me / ask me about?

**CHOs**

1. Have you started to recruit patients? How do you identify and register hypertensive and diabetic patients? Are there any challenges in recruiting the patients?
2. How do you treat or manage hypertensive or diabetic patients? Have you changed your practices? Why/ why not?
   1. Use of desk guide
   2. Prescription of drugs
   3. Advice on and monitoring of side effects
   4. Discussion of lifestyle interventions (smoking cessation, diet -salt, sugar and oil reduction and adherence)?
   5. Enablers and barriers for behaviour change
3. Can you tell me about how you communicate with patients during your consultations with them (time, challenges, trust)?
4. Do you interact with other colleagues in delivering hypertensive and diabetic care (who, for what reasons, referral)?
5. Can you tell me about patient adherence to follow-up appointments, medication and the associated enablers and barriers (e.g. drug supplies, patient affordability, doctor-patient trust, adherence support strategies)?
6. Can you tell me about the patient register and treatment cards? Are they easy to complete? Why / why not? Do you have any problems with them?
7. Can you tell me whether you think that patients are making any changes in their lifestyles? Why / why not? Which changes do you think are being made? Why / why not?
8. Do you receive any mentoring/supervisory services from the DHMT and RCGP/VSO doctors? Do you benefit from these?
9. Can you tell me about the training that you have attended?
   1. Do you feel that the training prepared you well for using this guide / delivering this intervention? Why / why not?
   2. Do you feel that you learned anything new in the training (information or skills)?
   3. What did you think about the training methods (presentations, role plays)? Did they help you to learn or do you think other methods would be better for you?
   4. Do you think the length of the training was appropriate? Should it be longer or shorter/ Why?
10. Were you involved with intervention adaptation and development? How do you feel about it?
11. In general, do you feel motivated to be part of the intervention? Why or why not?
12. Are there any other enablers and barriers of intervention development and delivery?

**DHMT member**

1. Were you involved with the intervention adaptation, development and care set up processes (treatment cards, drug supplies etc)? If yes, how? How do you feel about the implementation of the intervention (e.g. the working group process)? [follow up question for the last interview in Oct]
2. What is your impression of the intervention so far (progress, fidelity, quality)? Why do you have these impressions? (probe on barriers and enablers)
3. What do you think about the model (pilot/training of trainers followed by training of remaining CHOs for each section) organisation, content and impact of the training? [follow up question for the last interview in Oct]
4. Are you involved with the mentoring/supervisory trips to the CHCs? What do you do during these visits?
5. What needs to be improved in terms of guidelines, organization and the delivery of the intervention?
6. What would be needed to sustain and scale up this approach across the country?

**VSO/RCGP doctors/managers**

1. How do you feel about the process of the intervention adaptation, development and care set up processes (e.g. Treatment cards, drug supplies)? What are the enablers and barriers? [follow up question for the last interview in Oct]
2. What is your impression of the intervention so far (progress, fidelity, quality)? Why do you have these impressions? (barriers and enablers)
3. What do you think about the model (pilot/training of trainers followed by training of remaining CHOs for each section), organisation, content and impact of the training? [follow up question for the last interview in Oct]
4. How do you feel about the mentoring/supervisory trips to the CHC? What do you do during the visit?
5. What needs to be improved in terms of guidelines, organization and the delivery of intervention?
